# Influence of *Staphylococcus aureus* Strain Background on Sa3int Phage Life Cycle Switches

**DOI:** 10.3390/v14112471

**Published:** 2022-11-08

**Authors:** Carina Rohmer, Ronja Dobritz, Dilek Tuncbilek-Dere, Esther Lehmann, David Gerlach, Shilpa Elizabeth George, Taeok Bae, Kay Nieselt, Christiane Wolz

**Affiliations:** 1 Interfaculty Institute of Microbiology and Infection Medicine, University of Tübingen, 72074 Tübingen, Germany; 2 Fraunhofer Institute for Interfacial Engineering and Biotechnology IGB, 70569 Stuttgart, Germany; 3 Cluster of Excellence EXC 2124 “Controlling Microbes to Fight Infections”, University of Tübingen, 72074 Tübingen, Germany; 4 Institute for Bioinformatics and Medical Informatics, University of Tübingen, 72076 Tübingen, Germany; 5 Department of Microbiology, University of Würzburg, 97074 Würzburg, Germany; 6 Department of Microbiology and Immunology, Indiana University School of Medicine-Northwest, Gary, IN 46408, USA

**Keywords:** phage, virulence, induction, gene regulation, Staphylococcus, hemolysin

## Abstract

*Staphylococcus aureus* asymptomatically colonizes the nasal cavity of mammals, but it is also a leading cause of life-threatening infections. Most human nasal isolates carry Sa3 phages, which integrate into the bacterial *hlb* gene encoding a sphingomyelinase. The virulence factor-encoding genes carried by the Sa3-phages are highly human-specific, and most animal strains are Sa3 negative. Thus, both insertion and excision of the prophage could potentially confer a fitness advantage to *S. aureus*. Here, we analyzed the phage life cycle of two Sa3 phages, Φ13 and ΦN315, in different phage-cured *S. aureus* strains. Based on phage transfer experiments, strains could be classified into low (8325-4, SH1000, and USA300c) and high (MW2c and Newman-c) transfer strains. High-transfer strains promoted the replication of phages, whereas phage adsorption, integration, excision, or *recA* transcription was not significantly different between strains. RNASeq analyses of replication-deficient lysogens revealed no strain-specific differences in the CI/Mor regulatory switch. However, lytic genes were significantly upregulated in the high transfer strain MW2c Φ13 compared to strain 8325-4 Φ13. By transcriptional start site prediction, new promoter regions within the lytic modules were identified, which are likely targeted by specific host factors. Such host-phage interaction probably accounts for the strain-specific differences in phage replication and transfer frequency. Thus, the genetic makeup of the host strains may determine the rate of phage mobilization, a feature that might impact the speed at which certain strains can achieve host adaptation.

## 1. Introduction

*Staphylococcus aureus* is a major human pathogen but also colonizes and infects different animal species [1,2,3,4,5]. Transmission of *S. aureus* between humans and livestock is of particular concern as *S. aureus* isolates from farmed animals are often antibiotic-resistant [6]. Adaptation to the different mammalian hosts occurs largely through the acquisition/loss of mobile genetic elements. *S. aureus* has jumped between species many times, resulting in the dynamic gain and loss of host-specific adaptive genes, many of which are prophage encoded [5,7,8,9]. Most prominent is the repeated loss of the temperate Sa3int phages upon the jump of *S. aureus* from humans to different animals [10]. In several instances, the animal-adapted strain was transmitted back to humans, where it often reacquired Sa3int phages, emphasizing their important role in human colonization [1,8,9,10,11,12]. Up to 96% of human nasal isolates were observed to carry Sa3int phages integrated into the *hlb* locus, which encodes ß-hemolysin (Hlb), also named ß-toxin [13]. These phages carry genes that encode human-specific immune evasion factors [14] and other potential virulence factors [10]. The observation that Hlb is always functional after phage excision and that this process also occurs during human infections resulting in Hlb-positive sub-populations [15], indicates that under certain infectious conditions, Hlb is essential for bacterial survival.

Temperate staphylococcal phages belong to the family of *Siphoviridae*. The genomes of siphoviruses are typically organized into six functional modules: lysogeny, DNA replication, packaging, head, tail, and lysis. The evolution of phage lineages is driven by the lateral gene transfer of interchangeable genetic elements (modules), which consist of functionally related genes [8,13,16,17,18,19]. *S. aureus*-infecting siphoviruses have been classified according to polymorphisms of the integrase gene (*int*) [13,16,17,20]. The *int* type dictates chromosomal integration at cognate *attB* sites and is closely associated with the virulence gene content of the prophage [13]. However, due to the mosaic nature of *S. aureus* siphoviruses the distinct phage modules can show high homology between different Sa-Int phages. e.g., several open reading frames (ORFs) from the prototypic Sa3int phage Φ13 are homologous to those of PVL (Panton-Valentine leucocidin)-encoding Sa2int phages.

The molecular interactions between the *S. aureus* host and its temperate phages are largely unknown. The large number of phage genes encoding hypothetical proteins highlights how little is known about temperate phages and their influence on bacterial lifestyle switches. Likewise, we largely ignore which host factors influence the lysogenic-lytic cycle switch of temperate phages. Previous analysis of Sa2int phages revealed that the inducibility of the very same prophage can be significantly different when analyzed in diverse host genetic backgrounds [20]. Mobilization of the Sa2mw prophage from *S. aureus* strain MW2 or Newman was 100-fold higher than that from strain 8325-4.

Here, we investigated whether strain-specific features also impact the life cycle of Sa3int phages. For this, we constructed and integrated Sa3int phages into different phage-cured *S. aureus* strains (8325-4, SH1000, USA300c, Newman-c, and MW2c). We focused on two prototypic Sa3int phages, namely Φ13 and ΦN315. Φ13 is derived from the *S. aureus* reference strain 8325 of clonal complex (CC) 8. ΦN315 is derived from the methicillin-resistant strain N315 (CC5) and carries the *tarP* gene encoding for an alternative glycosyltransferase [21]. The strain background was found to impact phage transfer and replication. RNAseq analysis hint at specific interaction of host factors with the phage regulatory region located in the lytic module of the phage.

## 2. Materials and Methods

### 2.1. Growth Conditions

Unless otherwise stated, single-lysogens of *S. aureus* carrying Φ13kan (Tang et al., 2017) or ΦN315tet were used. *S. aureus* cells were grown in Tryptic Soy Broth (TSB) (Oxoid), 37 °C, 180 rpm. Precultures were supplemented with the appropriate antibiotics: kanamycin (KanA, 50 µg mL^−1^), tetracycline (Tet, 3 µg mL^−1^), erythromycin (Erm, 10 µg mL^−1^), chloramphenicol (Chloro, 10 µg mL^−1^), and streptomycin (Strep, 500 µg mL^−1^).

### 2.2. Strain Construction

Strains are listed in Supplementary Table S1, and oligonucleotides are listed in Supplementary Table S2.

#### 2.2.1. Selection of Strep Resistant Strains

*S. aureus* isolates were grown in TSB to OD_600_ = 0.7 and supplemented with Strep (500 μg mL^−1^). After 4 h of growth, serial dilutions were plated on TSB agar plates supplemented with Strep (500 μg mL^−1^). Resistant colonies were sub-cultured and growth compared to the parental strain. Only streptomycin-resistant clones that were not impaired in growth were used in this study.

#### 2.2.2. Generation of Phage Cured USA300 (USA300c)

Native prophages (Sa2int and Sa3int) of *S. aureus* strain USA300 were deleted using plasmid pKOR1 as described for *S. aureus* Newman [22]. In brief: a 2 kb DNA fragment containing the respective *attB* sequences was PCR-amplified with the following primers (for Sa2int prophage: primer627 and primer628; for Sa3int prophage: primer434 and primer435) and inserted into pKOR1 and mutagenesis performed as described.

#### 2.2.3. Generation of Phage Lysates and Lysogens

Phage lysates of Φ13kan or ΦN315tet were obtained after mitomycin C (500 ng mL^−1^) induction of 8325-4 Φ13kan or N315-ΦN315tet in liquid culture (OD_600_ = 0.7). After 2 h, 37 °C additional mitomycin C (500 ng mL^−1^) was added and supernatant collected after further incubation for 1 h (37 °C). Supernatants were filtered (0.45 µm pore size (Merck)), and phage titer was enumerated by plaque assay. Single-lysogens were obtained by incubation of 10^6^ phages with phage-cured *S. aureus* strains (10 mL) from the exponential growth phase (OD_600_ = 0.7) for 4 h, 37 °C. Selection of lysogens was performed on TSA plates containing KanA (50 µg mL^−1^) or Tet (3 µg mL^−1^), respectively. Single-lysogens were sub-cultivated four times, and phage integration at the cognate *att* site within *hlb* verified by loss of β-hemolysin production and PCR using oligonucleotides hlb675 and Sa3intfor.

#### 2.2.4. Construction of Phage ΦN315tet

Phage ΦN315 was labeled with a *tet* resistance cassette. *TetK was* amplified from plasmid pT181 using primer pair Tet2-F BamHI and Tet2-R BamHI. ΦN315 IEC specific overhangs were amplified using primer pair IEC:tet A+B and C +D. The three resulting fragments were fused using overlap extension PCR, ligated into plasmid pBASE6 [23], and cloned into *E. coli* DC10B. The vector was subsequently transferred into *S. aureus* N315 using electroporation and mutagenesis performed as described [23]. The mutation was verified by PCR and resulted in the replacement of phage-encoded *chp* and *scn* with the *tet* cassette.

#### 2.2.5. Construction of Phage Φ13kan-Δrep

Left and right flanking regions of prophage encoded replication factor (SAOUHSC_02217) were amplified via PCR using oligonucleotides PiMAYrepdelrev/repdelrev and repdelfor/PiMAYrepdelfor (Supplementary Table S2) and cloned into shuttle vector piMAY [24] by Gibson assembly in *E. coli* DC10B. The vector was transferred from *E. coli* DC10B into 8325-4 Φ13kan and MW2c Φ13kan via electroporation and mutagenesis performed as previously described [24]. Gene deletion was verified by sequencing PCR amplicons spanning the mutation site. Further, lysogens were checked for β-hemolysin negative phenotype on blood agar plates.

### 2.3. Phage Transfer Assay

Cultures of donor strains (single lysogens) and recipients (phage-cured, streptomycin-resistant) were grown to exponential phase (OD_600_ = 0.7), mixed at a ratio of 1:1, and co-cultivated for 4 h, 37 °C, 180 rpm. Single and mixed cultures were diluted in PBS, and colony-forming units (CFU) were determined on blood agar plates (Oxoid) and TSA agar plates containing single antibiotics (KanA 50 µg mL^−1^, Tet 3 µg mL^−1^, Strep 500 µg mL^−1^) and double antibiotics (KanA 50 µg mL^−1^, Strep 500 µg mL^−1^ or Tet 3 µg mL^−1^, Strep 500 µg mL^−1^), respectively. Phage transfer frequency was determined by CFU grown on double antibiotic-containing plates divided by CFU grown on TSA plates containing streptomycin (500 µg mL^−1^). Single colonies were analyzed for loss of Hlb synthesis and phage integration by PCR. All tested double-resistant colonies carried the *hlb* converting phage. Spontaneous resistance was monitored by plating donor and recipient strains on selective agar plates and was many magnitudes lower than the observed transfer rates.

### 2.4. Lysogenization Assay

Phage-cured derivatives of *S. aureus* isolates were grown to exponential growth phase (OD_600_ = 0.7), and 10^8^ bacteria per mL were infected with phages to a multiplicity of infection (MOI) of 0.1 or MOI 0.01 followed by incubation for 20 min or 4 h, 37 °C, 180 rpm. CFU was determined on blood agar plates and TSA agar plates containing either KanA or Tet for the selection of lysogens. Single colonies were picked on a blood agar plate to verify loss of ß-hemolysin activity. Lysogenization frequency was determined by CFU on antibiotic-containing plates divided by total CFU on blood plates.

### 2.5. Plaque Assay

Phage titer was determined by agar overlay method using strain LS1 as indicator strain. Indicator strains were grown to OD_600_ = 0.1 in TSB. In total, 100 µL bacterial culture was mixed with 3 mL liquid phage soft agar (Casaminoacids 3 g L^−1^, Yeast Extract 3 g L^−1^, NaCl 5.9 g L^−1^, Agar 7.5 g L^−1^) and poured on TSA plate. After solidification, dilutions of sterile-filtered phage lysates were dropped on the lawn and incubated at 37 °C to enumerate plaque-forming units (PFU).

### 2.6. Phage Adsorption Assay

Phage adsorption assays were performed as described [25] with slight modifications. In brief, 100 µL (3 × 10^6^ phages) were incubated with 3 × 10^8^ bacteria in 1 mL TSB for 10 min at room temperature under non-shaking conditions. Bacteria were pelleted (5000× *g*, 5 min), supernatant filtered (0.45 µm pore size (Labsolute)), and used for PFU determination.

### 2.7. Prophage Spontaneous Induction or Induction Using Mitomycin C

Single lysogens were grown to the exponential growth phase (OD_600_ = 0.7) and split into 10 mL aliquots. Aliquots were further incubated with and without subinhibitory concentrations of mitomycin C (300 ng mL^−1^) for 1 h. Supernatants were filtered (0.45 µm pore size (Merck)), PFU enumerated, and stored at −20 °C for qPCR. For absolute quantification of free phage DNA, 100 µL of phage lysates were incubated with Proteinase K (100 µg mL^−1^, AppliChem) for 1 h at 55 °C, followed by heat inactivation at 95 °C for 10 min. Phage DNA was quantified by quantitative PCR (qPCR) using SYBR Green qPCR Kit (QIAGEN) and primers circlefor and circlerev spanning the reconstituted *attP* site of the phage. For quantification, standard molecules were obtained by PCR using primers phi13circlefor and phi13circlerev. The amplicons were purified, and DNA concentration was determined using A_260_. For quantification of excised phages within bacteria, *attP* and the chromosomal *recA* (recAF1 and recA661) were quantified using bacterial pellets. Bacteria were mechanically lysed using zirconia/silica beads in a high-speed homogenizer (6500 rpm, Fastprep). Lysed pellets were boiled for 10 min in water bath and stored at −20 °C, and 1 µL of a 1:100 dilution (RNase-free water, Ambion) was used for qPCR.

### 2.8. Northern Blot Analysis and Preparation of RNA-Probes

Bacteria were grown to OD_600_ = 0.7, followed by 1 h incubation at 37 °C with or without mitomycin C (300 ng mL^−1^). In brief, the bacterial pellet was resuspended in TRIzol (Thermo Fisher Scientific, Waltham, MA, USA) and mechanically lysed using zirconia/silica beads in a high-speed homogenizer. For Northern blot analysis, RNA was isolated as recommended by the TRIzol manufacturer. Transcripts on Northern blots were hybridized with digoxigenin-labeled DNA probes generated by PCR (Supplementary Table S2). RNA probes were generated with specific primer pairs containing T7 promoter for in vitro transcription. In vitro transcription was performed with MEGAshortskript T7 kit following instructions with the exception that a nucleotide mix from Roche containing DIG-11-UTP was used for labeling of fragments with digoxigenin. For RNA-seq analysis, RNA from the aqueous phase was further purified using the ExpressArt^®^ RNA ready Add-on Kit for TRIzol extraction (AmpTec, Hamburg, Germany) with the following modifications. After loading the sample on an RNAready column, RNA was washed additionally with inhibitor removal buffer (5 M guanidine-HCl, 20 mM Tris-HCl pH 6.6, 37 % (*v*/*v*) EtOH). DNase digest of the sample was directly performed on the column.

### 2.9. TagRNAseq and RNAseq

RNA aliquots were subjected to tagRNA-seq [26]. Experiments were conducted by Vertis Biotechnologie AG. Library preparation on rRNA depleted RNA samples was performed as follows: first Illumina TruSeq sequencing adapter (CTGAAGCT) was ligated to RNAs containing a 5′monophosphate end (resulting from processing events and thereby representing so-called processed start sites—PSS) followed by treatment with TEX (Terminator Exonuclease, Lucigen) to remove unligated 5′P-ends. Next, RNA 5′Polyphosphatase (5′PP, Lucigen) was used to convert triphosphate groups at 5′-RNA ends to monophosphate 5′-RNA ends. Formed monophosphate ends were then tagged by ligation of a second Illumina TruSeq sequencing adapter (TAATGCGC) (representing transcription start sites—TSS). After fragmentation, an oligonucleotide adapter was ligated to the 3′ end of RNA fragments, and cDNA synthesis was performed using M-MLV reverse transcriptase. cDNA was PCR amplified within 16 cycles using high-fidelity DNA polymerase. cDNA was purified using Agencourt AMPure XP Kit (Beckman Coulter Genomics, Danvers, MA, USA). Last, the cDNA pool was single-read sequenced on an Illumina NextSeq 500 system using a 75 bp read length. Output read data were assigned to three different sets based on the tags from sequencing: read-files assigned to either transcriptional start site (TSS), read-files assigned to the processed start site (PSS), and unassigned read-files. The first two sets were used for transcription start site analysis, and the latter was used for expression analysis.

#### 2.9.1. Differential Expression Analysis of Phage-Encoded Genes Using tagRNA-seq

The reference genome of *S. aureus* 8325 (NCBI (NC_007795.1) was manually phage-cured, Φ13kan genome integrated and the sequence manually SNP-corrected based on resequencing of the 8325 strain [27]. Raw data files of reads (unassigned) were trimmed using the CLC genomics workbench (QIAGEN). Trimmed reads were mapped against the reference genome and then normalized for library depth and gene length, resulting in datasets containing expression values (RPKM-values).

Raw data and processed files containing RPKM-values are available at https://www.ncbi.nlm.nih.gov/geo/query/acc.cgi?acc=GSE214523 (accessed on 3 October 2022). Expression values were used for differential expression using the Wald test for statistical analysis. Significance was set to FDR-value of <0.05 and log2 fold change of lower than −1 or higher than +1. From the resulting datasets of the whole genome, the prophage genome was extracted and analyzed (Supplementary Table S4). Read mapping was visualized to the Φ13 genome using Integrated Genome Viewer.

#### 2.9.2. Determination of TSSs

To prepare the raw read data for TSS identification, reads were preprocessed and mapped, and a coverage per base was computed. For this, the RNA-seq analysis pipeline READemption version 0.5.0 [28] was used. All read samples were mapped to the respective reference sequence with the subcommand align, which integrates the mapper segemehl version 0.3.4 [29]. For the mapping, the following parameters were used: (--adapter AGATCGGAAGAGCACACGTCTGAACTCCAGTCAC, --processes 4, --segemehl_accuracy 95, --segemehl_evalue 5.0, --poly_a_clipping, --min_phred_score 20 --fastq, --progress). The subcommand coverage calculates one position-based coverage file, also called wiggle files, resulting in three file sets: the unnormalized raw wiggle files, files normalized by the total number of mapped reads (TNOAR) and multiplied by one million (mil_normalized), and files normalized by the total number of mapped read and multiplied by the lowest number of mapped reads taking all libraries in consideration (min_normalized). The min_normalized wiggle files were used for TSS calling. The TSS identification using the normalized wiggle files of the tagRNA-seq reads was conducted with TSSpredator 1.1 [30,31]. For all of the TSSpredator runs, the preset default parameters were used, except that matching replicates were set to 2. TSSpredator expects two types of reads, one from the so-called enriched library and one from the so-called normal or unenriched library. For the tagRNA-seq data, we used the TSS-labeled reads as the enriched libraries and the PSS-labeled reads as the normal control libraries. The experimental setup of this study used three strains and compared two conditions. Therefore, TSSpredator was run both with the strain-setup and condition-setup to analyze this data. For the cross-condition analysis, each strain was considered separately. For the cross-strain analysis, TSSpredator expects wiggle files normalized across all input libraries as input. For this, the lowest number of aligned reads over all replicates regarding both conditions was calculated, and then each library was multiplied by this minimum. From each TSSpredator run, the resulting MasterTables for each condition were combined manually, and the phage region was extracted (Supplementary Table S3). For the cross-condition analysis, each strain was considered separately. A detailed description of TSSpredator parameters, TSS classes, and output files can be taken from the user manual available at https://tsspredator20-rtd.readthedocs.io/en/latest/index.html (accessed on 3 November 2022).

### 2.10. Statistical Analysis

Statistical analyses were performed using GraphPad Prism software. Differences between the two groups were evaluated using Student’s t test. For multiple comparisons, statistical analysis was performed using one-way ANOVA (parametric) or Kruskal–Wallis test (non-parametric), with the Bonferroni test for parametric samples or Dunn’s test for non-parametric samples as a post hoc test. Differences at *p* < 0.05 were considered significant. All statistical analysis methods are based on independent biological replicates.

## 3. Results

### 3.1. Sa3int Phage Transfer during Co-Cultivation Depends on the Bacterial Host Strain

**Figure 1:**
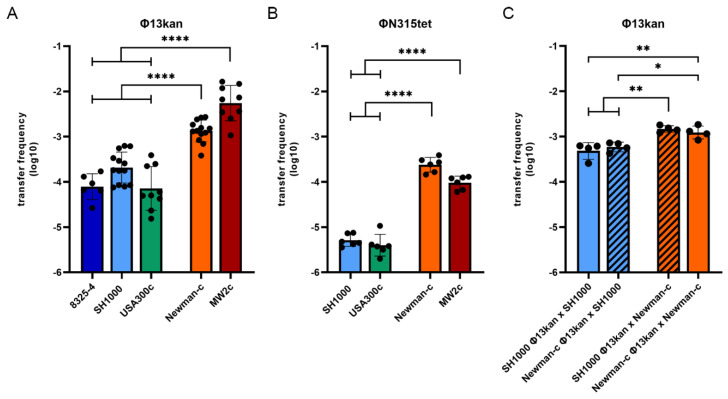
Phage transfer frequency of Φ13kan (**A**,**C**) or ΦN315tet (**B**) is strain-dependent. Lysogens were mixed with isogenic, phage-cured, streptomycin-resistant recipients 8325-4 (dark blue), SH1000 (light blue), USA300c (green), Newman-c (orange), or MW2c (dark red) at a 1:1 ratio (4 h co-culture in tryptic soy broth) (**A**,**B**) or with non-isogenic recipients (**C**). Phage transfer frequency was determined by calculating the ratio of CFU of double-resistant colonies (kanamycin/streptomycin for Φ13kan or tetracycline/streptomycin for ΦN315tet, respectively) divided by CFU on streptomycin (representing recipient). Values are independent biological replicates referring to mean ± SD. Statistical analysis was performed on log-transformed data using one-way ANOVA. * *p* ≤ 0.05, ** *p* ≤ 0.01, **** *p* ≤ 0.0001.

The Sa3int phage Φ13 is derived from the *S. aureus* reference strain 8325. This strain was previously cured of all phages, and the phage-cured derivative 8325-4 is widely used as a prototypic *S. aureus* strain in many genetic studies. To facilitate the analysis of the phage life cycle, a kanamycin (*kan*) resistance cassette was introduced at the 3′end of Φ13 [32]. To compare phage transfer/acquisition in different bacterial strains, Φ13kan lysogens were generated in different phage-free host strains: 8325-4, SH1000, MW2c, Newman-c, USA300c (Supplementary Table S1). Phage transfer was monitored after 4 h of co-culture of the Φ13kan single-lysogens with the isogenic, Strep-resistant and phage-free recipient under non-inducing conditions (Figure 1A). A high transfer rate was observed for strain MW2c and strain Newman-c, as enumerated by double resistance. Newly generated lysogens were Hlb-negative on blood agar plates, and phage integration into the *hlb* gene was verified by PCR using integration-specific oligonucleotides. Significantly lower phage transfer rates were observed for the 8325-4 or USA300c strain pairs as compared to Newman-c or MW2c. During the analyses, we observed that strain 8325-4 strain tended to aggregate during the incubation period. An *rsbU* repaired derivative of 8325-4 (strain SH1000) was described to form fewer aggregates [33]. To rule out any artifacts due to clumping, we also generated an SH1000 Φ13kan lysogen. This strain indeed did not aggregate but still showed a significantly lower phage transfer rate compared to Newman-c or MW2c. In summary, we could confirm that the host background significantly influences phage lifecycle and pinpoint high (Newman-c and MW2-c) and low (8325-4, SH1000 and USA300) phage transfer strains (Figure 1A).

To analyze whether the strain background similarly determines the transfer rate of other phages, we included phage ΦN315 derived from strain N315 in the analysis. The phage was labeled with a tet resistance cassette and mobilized into the same set of phage-cured strains. The phage transfer rate was lower compared to Φ13kan (Figure 1B). However, again strain Newman-c and MW2c exhibited higher phage transfer compared to the low-transfer strains SH1000 and USA300.

### 3.2. Strain-Dependent Differences in Sa3int Phage Transfer Are Determined by the Recipient

Strain 8325-4, SH1000, USA300, and Newman are assigned to the same CC 8 with no obvious restriction barrier [34]. MW2 belongs to CC1, and gene transfer between CC8 and CC1 strains is restricted due to different restriction/modification systems. Accordingly, phage transfer between CC8 strains and MW2 was found to be severely impaired (Supplementary Figure S1). We next analyzed whether transfer between low (SH1000) and high (Newman-c) transfer strains of the same CC is determined by the donor or by the recipient strain (Figure 1C). Strain Newman as recipient showed higher phage acquisition when incubated with either SH1000 Φ13kan or Newman-c Φ13kan. Strain SH1000 showed lower phage acquisition even when incubated with high transfer strain Newman-c Φ13kan. Thus, the different phage transfer rate among strains depends on the recipient.

### 3.3. Phage Adsorption Does Not Account for Strain Dependent Phage Integration

We speculated that strain-dependent phage transfer may be the result of differential phage adsorption. Wall-teichoic acids (WTA) function as conserved phage receptor for *S. aureus* phages [8]. This could be verified for Φ13kan since phage adsorption was not detectable in the WTA-deficient strain USA300 ∆*tagO* (Supplementary Figure S2). All tested wild-type strains showed high phage adsorption with only minor differences between strains. Small differences in phage adsorption did not correlate with the observed strain-dependent differences in lysogenicity efficiency. Thus, processes following initial phage adsorption are responsible for strain-specific differences in phage transfer and replication.

### 3.4. Strain Dependent Sa3int Lysogenization and Replication

**Figure 2:**
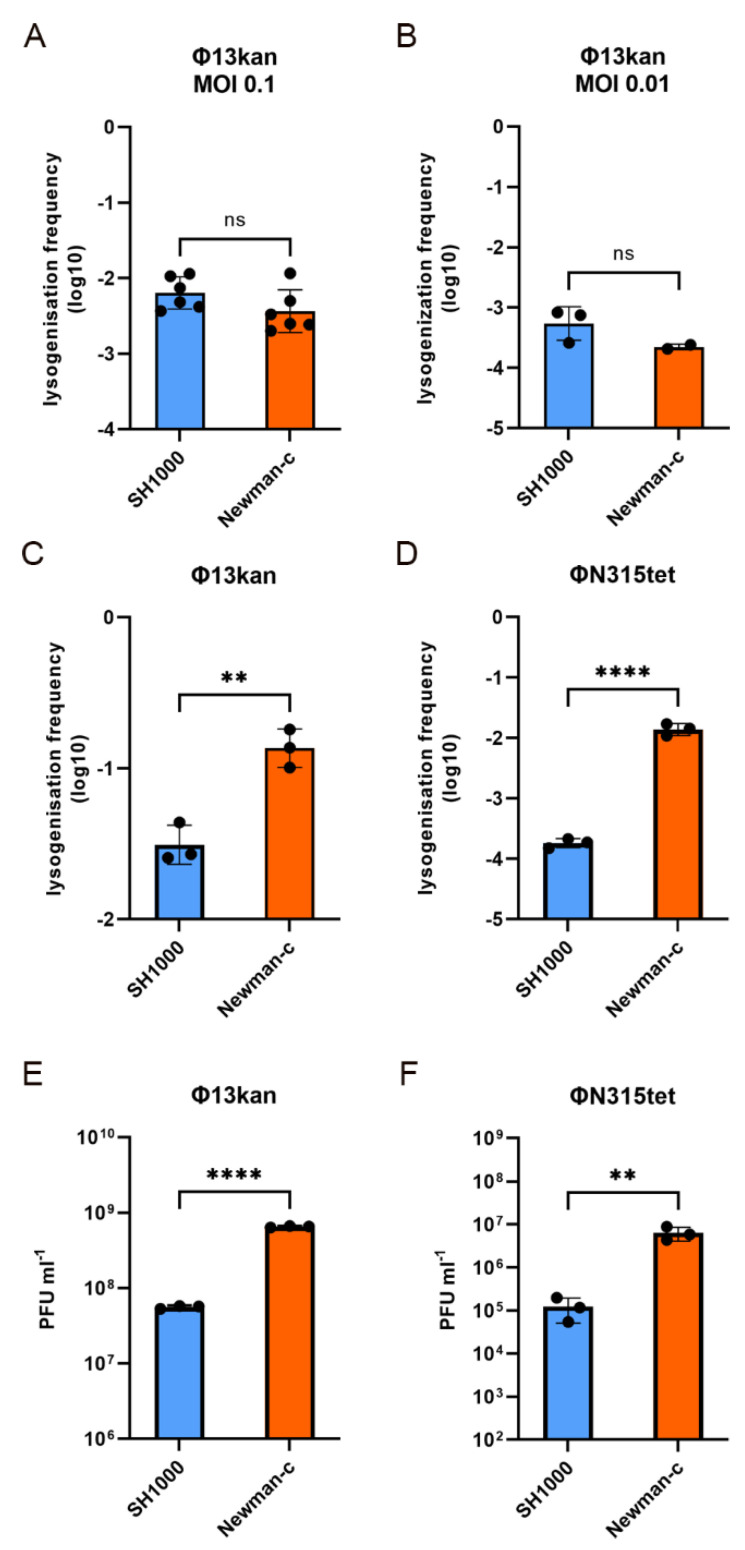
Strain-dependent lysogenization and replication of Φ13kan and ΦN315tet. Lysogenization of phage-cured SH1000 (blue) and Newman-c (orange) with Φ13kan (**A**–**C**,**E**) or ΦN315-tet (**D**,**F**) during exponential growth. Phages and phage-cured recipient bacteria were co-cultured to investigate lysogenization rates. Co-incubation was performed for 20 min at 37 °C and MOI of 0.1 (**A**) or 0.01 (**B**) or for 4 h at 37 °C and MOI of 0.01 (**C**–**F**). Lysogenization rates were determined by CFU on TSA plates containing kanamycin or tetracyclin (representing lysogenized colonies)/CFU on blood-agar plates. Replication of Φ13kan (**E**) and ΦN315tet (**F**) was determined by enumerating phage particles in sterile filtrated supernatant by plaque assay 4 h post phage infection. Values are independent biological replicates referring to mean ± SD. Statistical analysis was performed using t-test (**A**,**B**) and one-way ANOVA of log-transformed data (**C**–**F**). Data are back-transformed for visualization of phage titers (**E**,**F**). ** *p* ≤ 0.01, **** *p* ≤ 0.0001.

We next analyzed whether the various bacterial recipients differed in phage integration and/or replication rate. Newman-c lysogens were treated with mitomycin to induce prophage excision, and phage titer was enumerated by plaque assay. Phages were incubated with recipient strains, and phage integration was enumerated by the phage resistance marker. After 20 min co-incubation of Φ13kan with recipient strains, a similar fraction of SH1000 and Newman-c bacteria became lysogens (Figure 2A,B). This indicates that phage integration is equally efficient in both strains. However, after prolonged incubation (4 h), significantly more Φ13kan (Figure 2C) and ΦN315tet (Figure 2D) lysogens were recovered in the high transfer strain Newman-c compared to the low transfer strain SH1000. We assumed that this is due to enhanced phage replication in strain Newman-c. To monitor phage replication, free phage titers were enumerated after the 4 h incubation period. Significantly higher phage replication of both phages (Φ13kan and ΦN315tet) was observed after phage infection of strain Newman-c compared to strain SH1000 (Figure 2E,F). The phage titer of Φ13kan increased 650-fold in strain Newman-c and only 55-fold in strain SH1000.

Thus, the integration efficiency seems not to differ between strains. However, in the high transfer strain, Newman-c phage replication is enhanced. The higher phage replication, in turn, increases the chance of phage integration, as seen in the later time points of infection.

### 3.5. Phage Gene Expression Is Dependent on the Host Strain Background

**Figure 3:**
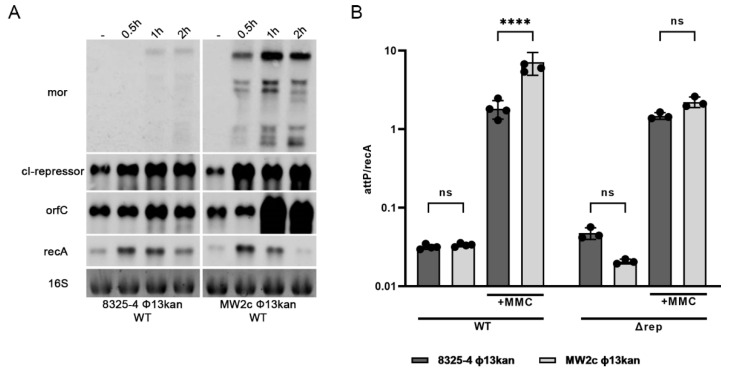
Phage replication is strain-dependent. Transcriptional analysis of phage-related genes (cI, mor, orfC) and chromosomally encoded recA under non-inducing and phage-inducing conditions (**A**). 8325-4 Φ13kan and MW2c Φ13kan were grown to OD_600_ = 0.7 and treated with mitomycin for 0.5 h, 1 h, or 2 h) or without mitomycin (1 h). RNA was hybridized with digoxygenin labeled DNA probes. Phage induction/replication in wild type (8325-4 Φ13kan, MW2c Φ13kan) and replication-deficient derivatives (Δ*rep*) under non-inducing and phage-inducing (1 h mitomycin, MMC) conditions (**B**). Phage excision/replication was enumerated as the ratio of excised, circularized phage copies (attP) per copy of bacterial chromosome (recA) as quantified by qPCR. **** *p* ≤ 0.0001.

Most of the genes carried by Φ13 encode proteins of unknown functions. Recently, a regulatory switch region was identified in the Φ13 genome [35]. The region is composed of a CI coding repressor gene (*cI*) and a divergently transcribed *mor* (modulator of repression) gene. We monitored the transcription of lysogenic genes presumably initiating from the *cI* promoter and of the lytic genes initiating from *mor* by Northern blot analysis in MW2c- and 8325-4 Φ13kan lysogens (Figure 3A). As expected, transcription of lytic genes (*mor*) was only detected after mitomycin treatment. One major transcript was detected, representing co-expression of *mor* with downstream lytic genes. Several additional bands were also visible, indicating the processing of the transcript. The expression of these lytic genes was mainly detectable in the MW2-c Φ13kan strain. This is consistent with higher replication observed in high phage transfer strain, i.e., Newman (Figure 2E). Higher phage replication in the MW2 background could be verified by quantification of phage copy numbers after mitomycin treatment via qPCR (Figure 3B). Phage replication also explains why the expression of genes within the lysogenic module, such as *cI* and *orfC,* was also increased after mitomycin treatment. Thus, multi-copy effect after phage replication impedes the interpretation of these results.

Φ13 is likely induced following DNA damage-mediated RecA activation and subsequent CI autocleavage [35]. We speculated that MW2 might be more sensitive to RecA activation/SOS response compared to 8325-4. Therefore, we compared the induction of the SOS gene *recA* (preceded by a canonical LexA binding motif) in both strains. No difference in mitomycin-induced *recA* expression was observed between strains. In summary, the Northern blot analysis supported the hypothesis that RecA-independent bacterial factors promote phage replication in high-transfer strains.

### 3.6. Induction of Φ13kan-Δrep Is Not Strain Dependent

To exclude multi-copy effects, we constructed replication-deficient mutants (Φ13kan-Δrep) in different bacterial strain backgrounds. In such mutants, the phage can excise but not replicate (Figure 3B). No phage particles were detectable by plaque assays. Calculation of the excised prophage genomes per bacterial cell was performed by absolute quantification of *attP* in relation to *recA* (as a proxy for bacterial genome copy numbers) via qPCR. After mitomycin treatment, roughly one excised phage copy per bacterial genome was produced with no significant differences between strains. This indicates that phage induction is not significantly different between strains.

### 3.7. Transcriptional Start Site Prediction of Φ13kan-Δrep

**Figure 4:**
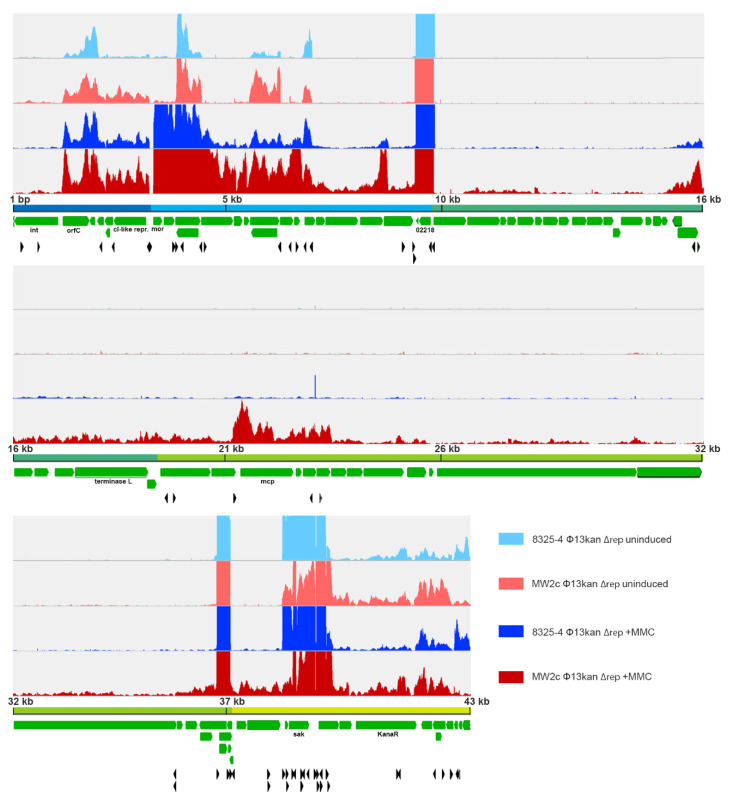
Visualization of predicted and enriched TSS and transcriptional profile of extracted Φ13 prophage region. Reads mapping to the phage genome are visualized for MW2c- and 8325-4 Φ13kan-∆rep strains under uninduced (light red and light blue, respectively) and induced (1 h mitomycin C treatment; dark red and dark blue) conditions from a representative sample out of 3 replicates. The maximum for the Y-axis in each panel is 200 counts. Protein coding genes are depicted in green and predicted TSS (Table S3) by black arrowheads.

Besides the CI/Mor regulatory switch region, additional regulatory elements on the phage genome are likely involved in the phage-host cross-talk. So far, such putative regulatory elements and additional phage promoters are ill-defined. Therefore, we aimed to dissect phage transcriptional units in the replication-deficient lysogens 8325-4 Φ13kan-∆rep and MW2c Φ13kan-∆rep using tagRNA-seq [26] followed by comparative analysis with putative TSSs as predicted by TSSpredator [31] (Figure 4, Supplementary Table S3).

We detected a total of 57 transcriptional start sites. For prediction, at least two replicates had to agree on the position of a TSS. The expression of corresponding genes is shown in Figure 4 and Supplementary Table S3. In general, transcription followed the expected orientation, namely leftwards in the lysogenic module and rightwards in the lytic/structural modules.

**Figure 5:**
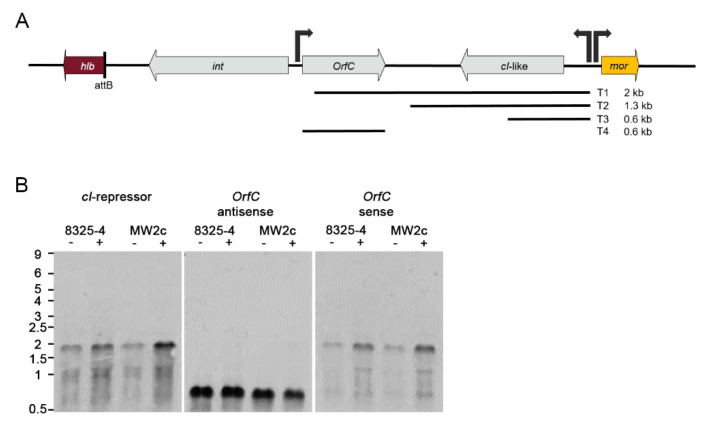
Transcriptional organization of the lysogenic modules of Φ13. Schematic representation of the lyosegenic module (**A**) of Φ13. Transcriptional units were visualized by hybridization of total RNA with digoxigenin-labeled RNA probes (**B**). Replication-deficient derivatives (Δrep) of 8325-4 Φ13kan and MW2c Φ13kan were grown to OD_600_ = 0.7 and treated with (+) or without (-) mitomycin (1 h).

The analysis also revealed several noncontiguous operons (operons containing a gene(s) that is transcribed in the opposite direction to the rest of the operon) (Figure 4, Supplementary Table S3), which were only recently acknowledged to play a role in gene regulation [36]. For instance, a single gene transcript (S861) located next to *ant* (putative anti-repressor, SAOUHSC_02229) is part of a lytic transcript but is also transcribed in anti-sense direction towards the lysogenic module (SAOUHSC_02232) (Supplementary Table S4). A noncontiguous operon was also detected in the lysogenic module. *orfC* located between *int* and the putative phage repressor *ci* is transcribed opposite to the expected lysogenic direction (Figure 5A). *CI* transcription is initiated between *mor* and *cI* from the predicted promoter identified previously [35] (Figure 5). Northern blot analysis using strand-specific RNA probes confirmed that a transcript spanning c*I* and *orfC* is simultaneously detectable with a smaller *orfC* transcript starting from the opposite strand (Figure 5B).

Thus, transcriptional regulation of phage genes seems highly complex, and gene expression is controlled at several levels. While most of the 57 TSSs were common to both strain backgrounds, strain-specific TSSs were also identified. In total, 14 of the predicted TSS are specific to 8325-4 Φ13kan (5 TSS: uninduced, 8 TSS: +MMC, 1 TSS: both conditions) and 14 TSS to MW2c Φ13kan (1 TSS: uninduced, 12 TSS: +MMC, 1 TSS: both conditions).

### 3.8. Strain-Dependent Gene Expression of Φ13kan-Δrep

**Figure 6:**
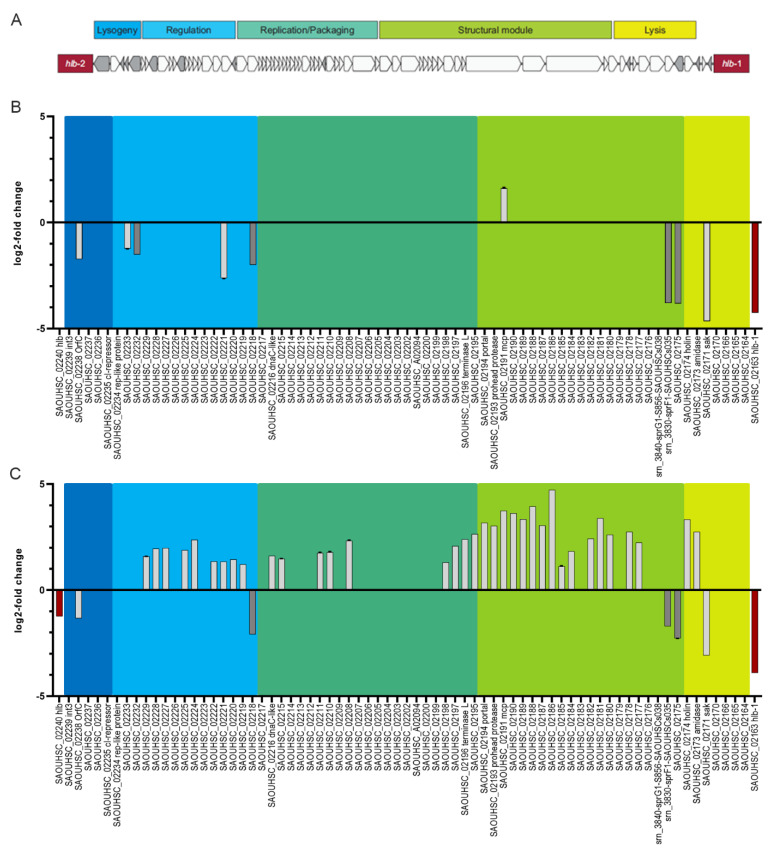
Module organization of Φ13kan (**A**). Comparison of Φ13kan-Δrep gene expression between MW2- and 8325-background under uninduced conditions (**B**) and after mitomycin induction (**C**). Only genes with significant differences in gene expression are shown (FDR-value < 0.05; log2fold change >+1 and <−1). Negative values represent higher expression in 8325 background; positive values represent higher expression in MW2 background.

Phage gene expression of Φ13kan-Δrep in 8325-4 and MW2c background was quantified by RNA-seq under non-induced and induced (1 h, mitomycin treatment) conditions (Supplementary Table S4). Under uninduced conditions, only nine phage genes were differentially expressed between both strains (Figure 6B). *OrfC* was expressed at higher levels in the 8325-background compared to the MW2-background. This is also evident in the Northern blot analysis of the Φ13kan-Δrep mutant (Figure 5B). The noncontiguous genes SAOUHSC_02232 and SAOUHSC_02218 were also more highly expressed in the 8325-4 background. Interestingly, these two genes of unknown function are transcribed in lysogenic direction although localized within the lytic phage region. Additionally, IEC genes showed higher expression in 8325 compared to MW2 (e.g., *sak*, coding for staphylokinase, and the TA-system sprGF1/sprF1). The expression of *sak* was previously reported to be dependent on the host strain background, with expression being lower in MW2 compared to RN6390 (a derivative of 8325) background [20]. This is in agreement with previous work showing that Sa3int-encoded virulence genes are expressed independently from the phage lifecycle [10]. *Mcp* is the only gene that was found to be significantly higher expressed in strain MW2c than in 8325-4 under non-inducing conditions.

We next compared strain-dependent phage gene expression after mitomycin treatment (1 h), revealing a more profound difference in phage gene expression between the two strains. Most of the prophage genes either in 8325 (40 out of 73) and MW2 (56 out of 73) were significantly upregulated after mitomycin C treatment (Supplementary Table S4). However, upregulation was more pronounced in the MW2 background; 35 prophage-encoded genes were expressed at significantly higher levels in MW2 compared to the 8325 background (Figure 6C). Interestingly, no differences in the expression of *cI* and *mor* were observed between strains. Instead, most differences appeared in later genes assigned to regulation (blue), replication/packaging (dark green), and structural module (light green). Several TSSs were predicted within the lytic module, assuming that the expression of gene modules is dependent on different promoters (Figure 4 and Supplementary Tables S3 and S4), which are subject to strain-specific regulation.

Thus, the expression of lytic genes of prophage Φ13kan-∆rep is more pronounced in the MW2 background compared to 8325 and probably accounts for the higher phage particle number observed in the MW2 background.

## 4. Discussion

Here, we demonstrated that the lifecycle of *S. aureus* prophage is influenced by bacterial host factors. Replication of the *hlb* converting phages Φ13 and ΦN315 was significantly enhanced in the Newman and MW2 strains compared to derivatives of the 8325 lineage or USA300. The higher replication rate correlated with higher spontaneous phage transfer events during co-culture. A similar dependence of phage replication on the host background was previously shown for unrelated *pvl*-carrying ΦSa2mw phage [20]. The strain dependency is not due to differences in phage absorption (Supplementary Figure S2), phage integration (Figure 2), excision, or *recA* transcription (Figure 3). Interference with other phages could also be ruled out since all experiments were performed in single-lysogens.

To obtain more insights into the molecular mechanisms of the predicted phage–host interaction, we performed tagRNA-seq and determined the TSSs located on the Φ13kan-∆rep phage and compared the expression of phage genes in 8325-4 and MW2c background. The decision between phage lifestyles is made by phage-encoded genetic switches best studied in phage λ of *Escherichia coli*. The transcriptional repressors CI and Cro can repress each other and compete for the same operator. Phage repressor CI represses transcription of lytic genes, whereas Cro relieves repression, thus facilitating the lytic life cycle. A CI/Cro-like switch region can be identified in ΦN315 and ΦSa2mw2. However, in Φ13, the switch region is composed of an autocleavable CI repressor and a Mor homolog [35,37]. We confirmed the previously identified promoter regions as well as mitomycin sensitivity of Φ13. The CI repressor, which is expressed from the lysogenic promoter PR, represses the lytic promoter initiating *mor* expression. The small repressor MOR, first identified in lactococcal phages, functions as an anti-repressor of CI by protein-protein interaction but, on its own, does not directly influence transcription [37,38,39]. Thus, *S. aureus* phages can carry either CI/Mor (Φ13)- or CI/Cro-like switches (ΦN315, ΦSa3mw) but nevertheless show similar strain dependency. It is thus unlikely that the proposed host factors influencing the phage life cycle target the canonical switch regions. Indeed, the expression of *cI* and *mor* genes was not significantly different between strains. Analysis of promoter fusions in *B. subtilis* also indicated that the decision switching by the minimal switch region of Φ 13 does not require *S. aureus*-specific host factors [35]. This is also in line with the observation that phage integration/excision seems to be independent of the strain background and likely only determined by RecA activity.

Nevertheless, replication and gene expression were elevated in the MW2 strain background, suggesting specific regulation downstream of the switch region. The TSS analysis points to many additional promoter regions that could be subject to gene regulation. The phage-derived mRNA landscape is further complicated by additional RNA processing sites. RNA processing might also be tightly controlled and functionally important [40,41]. Additionally, sRNAs or post-transcriptional regulation via, e.g., anti-termination [42] may be involved. Moreover, our analysis revealed several noncontiguous operons. Such a genetic arrangement was proposed to lead to mutual regulation of the expression of overlapping transcripts and to provide an additional strategy for coordinating the expression of functionally related genes within an operon [36].

Some TSSs were detected only in one strain background. Genes coding for structural phage proteins are more highly expressed in MW2 than in 8325-4. Thus, these TSSs are likely targets for host-dependent regulation. Post-excision regulation of phage replication and assembly, processes that are important to finalize the phage lytic phase, may also be a crucial determinant of the lysogenic-lytic cycle switch. Thus, under certain circumstances, the phages may just excise, leading to the reconstitution of the intact *hlb* gene in the absence of complete phage assembly and host cell lysis. Such a process is reminiscent of the recently described process termed active lysogeny [43]. *L. monocytogenes* strain 10403S harbors a prophage in its *comK* gene. During infection of macrophage cells, the prophage lytic pathway is induced, but the phage lytic response is arrested, preventing the expression of the late genes. A phage-encoded LlgA transcription regulator (LlgA) and a DNA replicase are proposed to support the phage adaptive behavior [44]. No LlgA homolog could be identified, but functionally similar elements may be present in the *S. aureus* phages. The data strongly indicate that host factors interfere with such putative regulatory phage regions. Recently discussed xenogenic silencing factors might be involved in such a balance [45]. They promote tolerance of foreign genetic material and may play an important role in maintaining the lysogenic state. Those discovered so far are small, nucleoid-associated proteins that recognize and bind AT-rich DNA stretches (H-NS in Proteobacteria, MvaT/U in *Pseudomonas* species, Rok in *Bacillus subtilis* and Lsr2 in Actinobacteria). Of note, none of these factors is present in *S. aureus*. The phage-encoded virulence genes are known to be under the control of bacterial virulence regulatory systems such as the quorum sensing system, Agr, or the SaeRS two-component system [46]. From genome comparison between the low and high transfer strains, no obvious difference in known regulatory circuits, such as quorum sensing or transcriptional factors, was evident. Nevertheless, our data indicate that also phage structural genes are tightly controlled, although the mechanism remains to be determined.

Epidemiological data strongly indicate that Sa3int phages have co-evolved with the *S. aureus* host to facilitate the adaptation of the species to the human host. Adaptation is likely mediated by the phage-encoded virulence factors, which are specific to humans [10]. Of note, most *S. aureus* strains lack major phage defense systems such as CRISPR/Cas, cyclic-oligonucleotide-based anti-phage signaling systems [47] or phage-resistance mediating retrons [48]. This indicates that the species has likely evolved to tolerate phages and that the phages, to a large extent, are probably beneficial to the bacterial host. However, the phages remain highly mobile to relieve expression of the interrupted *hlb* gene when needed, e.g., during infection [15,49]. This may occur either in the form of “active lysogeny” or prophage loss. Such switches must be controlled through firm molecular interactions between bacterial and phage factors. The postulated bacterial factors are highly strain-specific, and certain *S. aureus* strains may be more prone than others to support either a lysogenic or a lytic life cycle. Thus, the genetic makeup of the host strains may determine the rate of phage mobilization during infection, a feature that might determine the speed at which certain strains can achieve host adaptation.

## Data Availability

RNAseq raw data and processed files containing RPKM-values are available at https://www.ncbi.nlm.nih.gov/geo/query/acc.cgi?acc=GSE214523 (accessed on 3 October 2022).

## Supplementary Materials

The following supporting information can be downloaded at: https://www.mdpi.com/article/10.3390/v14112471/s1, Figure S1: Phage transfer between CC8 and CC1; Figure S2: Phage adsorption; Table S1: Strains used in this study; Table S2: Oligonucleotides used in this study; Table S3: TSS prediction and corresponding gene expression; Table S4: Differential gene expression analysis of extracted prophage genomic region.
