# Supplementary material for: Influence of Staphylococcus aureus Strain Background on Sa3int Phage Life Cycle Switches
Source: Viruses. 2022 Nov 8;14(11):2471. doi: 10.3390/v14112471 (PMC9694928; doi:10.3390/v14112471)
Supplement: Supplementary file 1 [file viruses-14-02471-s001.zip › Supplementary Figures.pdf]

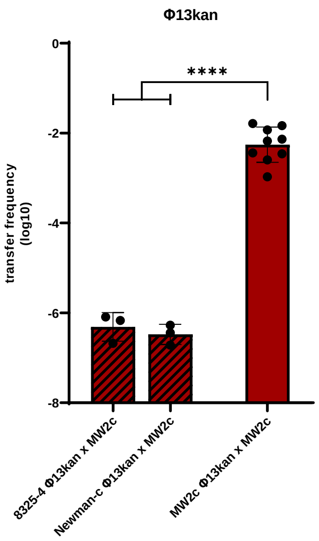

Figure S1. Phage transfer frequency between CC8 donors (8325-4 Φ13kan, Newman-c Φ13kan) and CC1 (MW2c) recipient strain is restricted. Lysogens were mixed with phage-cured, streptomycin-resistant recipients MW2c at a 1:1 ratio (4 h coculture in tryptic soy broth). Phage transfer frequency was determined by calculating the ratio of CFU of double-resistant colonies (kanamycin/streptomycin) divided by CFU on streptomycin (representing recipient). Values are independent biological replicates referring to mean ± SD. Statistical analysis was performed on log-transformed data using one-way ANOVA.

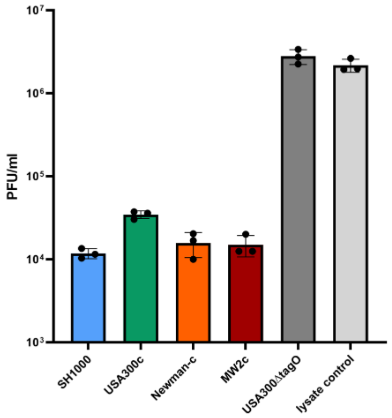

Figure S2. Phage adsorption. 10^6 Φphi13kan phage particles were incubated with 10^8 phage cured bacteria or a WTA deficient *tagO* mutant for 10 minutes. Unbound phages in the filtered supernatants were enumerated by plaque assays. Values are independent biological replicates with mean ± SD.
