# Supplementary material for: Influence of Staphylococcus aureus Strain Background on Sa3int Phage Life Cycle Switches
Source: Viruses. 2022 Nov 8;14(11):2471. doi: 10.3390/v14112471 (PMC9694928; doi:10.3390/v14112471)
Supplement: Supplementary file 1 [file viruses-14-02471-s001.zip › Supplementary Tables.pdf]

**Table S1.** Strains used in this study

| Bacterial strain | Clonal complex (CC) | Property | Origin |
|---|---|---|---|
| 8325-4 | CC8 | Phage-cured | Dorte Frees, University of Copenhagen, Denmark |
| 8325-4-Strep | CC8 | Phage-cured, resistant against streptomycin | This study |
| 8325-4Φ13-kana | CC8 | Single-lysogen | This study |
| 8325-4ΦN315-tet | CC8 | Single-lysogen | This study |
| 8325-4Φ13-kanaΔrep | CC8 | Single-lysogen carrying replication-deficient mutant | This study |
| SH1000 | CC8 | Phage cured | Susanne Engelmann, TU Braunschweig, Germany |
| SH1000-Strep | CC8 | Phage-cured, resistant against Streptomycin | This study |
| SH1000Φ13-kana | CC8 | Single-lysogen | This study |
| SH1000ΦN315-tet | CC8 | Single-lysogen | This study |
| USA300c | CC8 | Phage-cured | This study |
| USA300c-Strep | CC8 | Phage-cured, resistant against Streptomycin | This study |
| USA300cΦ13-kana | CC8 | Single-Lysogen | This study |
| USA300cΦN315-tet | CC8 | Single-lysogen | This study |
| Newman-c | CC8 | Phage-cured | T. Bae et al., 2006 |
| Newman-c-Strep | CC8 | Phage-cured, resistant against Streptomycin | This study |
| Newman-cΦ13-kana | CC8 | Single-Lysogen | This study |
| Newman-cΦN315-tet | CC8 | Single-Lysogen | This study |
| MW2c | CC1 | Phage-cured | Tang et al., 2017 |
| MW2c-Strep | CC1 | Phage-cured, resistant against Streptomycin | This study |
| MW2cpΦ13-kana | CC1 | Single-Lysogen | This study |
| MW2cΦN315-tet | CC1 | Single-lysogen | This study |
| MW2cΦ13-kanaΔrep | CC1 | Single-lysogen carrying replication-deficient mutant | This study |
| USA300_Δ*tagO* | CC8 | WTA-deficient isolate used as control for adsorption-assay | Wanner et al., 2008 |
| *E. coli* DC10B | - | Used for cloning procedures | Monk et al.,2012 |

**Table S2.** Primer used in this study

| Name | Sequence |
|---|---|
| circlefor | TTTTATTTTATATGGGGTATTATTGA |
| circlrev | GTGTATTCTCATTTGTTAGAAGAAAA |
| hlb675 | GCTATCATTATCGAATCCAC |
| hlb258 | |
| IEC::tet_A | GACGAATTCGTGAAAAGGGTTGTTTATGGGGC |
| IEC::tet_B | CTTATATTTTGTTCTAGGATCCCTGTGAATAGTCATAGGCGTCCATACATAATC |
| IEC::tet_C | GAGTTTTTAGAACAAGGATCCGGTAAAGAAAGTGTTAGGTTACTAGGCCACTTAAC |
| IEC::tet_D | CTCGAGCTCCCCTGGATTCAACTTAATTACAAAGG |
| phi13cIDIGfor | TCATACTTCGGATTTAGAGATACC |
| phi13cIDIGrev | CGAAACCTTATCAAAAGAAACTAGG |
| phi13croDIGfor | CGGTAAAGTTGGTTGGAA |
| phi13croDIGrev | ATTGGAGTGGCGTTGATT |
| phi13sieDIGrev | GAAATCGCTACCAGCTGA |
| phi13sieDIGfor | CGCTTCTTCTTACAGGAGTT |
| Primer434 | GGGGACAAGTTTGTACAAAAAAGCAGGCTCCGTTCACAGTGATTGTGTATGG |
| Primer435 | GGGGACCACTTTGTACAAGAAAGCTGGGTGCCTGCTACATAGAATGTAGTAGG |
| Primer627 | GGGGACAAGTTTGTACAAAAAAGCAGGCTCAATTACATCATCAACTGTATTGTC |
| Primer628 | GGGGACCACTTTGTACAAGAAAGCTGGGTGATGCGTTGAGTAAACTGATTAC |
| recAF1 | GCTCAAGCATTAGGCGTAGAT |
| recA661 | ATTTTAATGCACGTCCACCTGG |
| sa3intfor | GAAAAACAAACGGTGCTAT |
| sa3intrev | TTATTGACTCTACAGGCTGA |
| phi13circlefor | TCTAGCTTTTGGGGTGTACATTCC |
| phi13circlerev | GCTTTGAAATCAGCCTGTAGAG |
| pIMAYrepdelrev | TCGATAAGCTTGATATCGACTAGAAAACGGATATCCACT |
| pIMAYrepdelfor | GATCCCCCGGGCTGCAGGTCTCGCTCCCTGAAATCGTC |
| repdelfor | AAATGGCAACAGAAACACTTTTTGGCAGT |
| repdelrev | GTGTTTCTGTTGCCATTTCGTTATCTCCTTTCTG |
| Tet2-F BamHI | TTCACAGGGATCCTAGAACAAAATATAAG |
| Tet2-R BamHI | TCTTTACCGGATCCTTGTTCTAAAAACTC |

**Table S3. TSS prediction and corresponding gene expression.** Table includes summarized information on predicted TSSs obtained from TSSpredator (https://uni-tuebingen.de/fakultaeten/mathematisch-naturwissenschaftliche-fakultaet/fachbereiche/informatik/lehrstuehle/integrative-transkriptomik/software/tsspredator/). Detailed description of included information are given in the user guide for TSSpredator V1.1 (https://itnc.informatik.uni-tuebingen.de/index.php/s/en3s7fegaCzWQQy). Information contained in Mastertable are given for each predicted TSS (listed in rows). TSS number (Column A), neighbouring gene (Column B) or potential gene according to direction  and position (Column O). Sequence -50 nt upstream + TSS (51nt) contains the base of the TSS and the 50 nucleotides upstream of the TSS (Column E). Column F-M indicate under which conditions the predicted TSS was enriched and the gene expression of the corresponding gene according to the direction and position of the TSS.

**Table S4. Differential gene expression analysis of extracted prophage genomic region.** Table contains combined data obtained from CLC genomics workbench (QIAGEN) including expression analysis for prophage genomic region for comparison of MW2 versus 8325 gene locus (**Column A**). Expression values of uninduced condition of MW2vs8325 comparison is marked in yellow (**Column D-F**), expression values for induced condition MW2vs8325 comparison are marked in green (**Column G-I**). Expression values for comparison of condition (induced versus control) are marked in dark blue for 8325 (**column J-L**) and dark red for MW2 (**Column M-O**). RPKM values are listed in Column P-AA and marked in light blue for 8325 and red for MW2 for both conditions.
